# Pregnancy and Dental Caries

**Published:** 1896-03

**Authors:** 


### Pregnancy and Dental Caries.

Dental caries is a disease characterized by molecular disintegration of the normal constituents of the teeth, and is probably more liable to occur during pregnancy; it is caused by the same processes which produce lactic acid, which latter in turn decalcifies the enamel and exposes the dentine. There is evidence to prove also that the saliva is more acid during the period of gestation than at other times; which, if true, is probably due to changes in the blood whereby its alkalinity is diminished. The analogy between this and the lithemic condition is striking.

It is improbable that lime salts are abstracted from the teeth to supply the needs of the growing fetus; more than enough phosphates are ingested to supply the needs of both mother and child, hence maternal teeth do not suffer from lack of nutrition; again, during gestation osteophytes are found, evidencing an excess of lime salts in the system.

Vomiting of pregnancy, while it may to some extent aid, cannot be considered a potent factor in the production of dental caries; neither can neglect of the teeth during pregnancy be proved to be more prevalent than at other times.—*Peterson.*

